# Time trade-off with someone to live for: impact of having significant others on time trade-off valuations of hypothetical health states

**DOI:** 10.1007/s11136-021-03026-6

**Published:** 2021-10-30

**Authors:** Tonya Moen Hansen, Knut Stavem, Kim Rand

**Affiliations:** 1 Division for Health Services, Norwegian Institute of Public Health, Oslo, Norway; 2 Health Services Research Unit, Akershus University Hospital, Nordbyhagen, Norway; 3 Institute of Clinical Medicine, University of Oslo, Oslo, Norway; 4 Medical Division, Department of Pulmonary Medicine, Akershus University Hospital, Lørenskog, Norway; 5 Maths in Health B.V, Rotterdam, The Netherlands

**Keywords:** Health state valuation, Time trade-off, EQ-5D, Health-related quality of life

## Abstract

**Background:**

The TTO task involves giving up life years, i.e. living a shorter life, to avoid an undesirable health state. Despite being a hypothetical task, some respondents take other life factors into account when completing the task. This study explored the effect of having children and/or a partner on TTO valuations of hypothetical EQ-5D-5L health states in a valuation study of the general population.

**Methods:**

The study used TTO data collected in a Norwegian EQ-5D-5L valuation study in 2019–2020, by one-to-one pc-assisted interviews following the EQ-VT protocol. We used regression modelling to determine the effect of significant others (having children or a partner) on disutility per health state from the TTO valuations.

**Results:**

430 respondents were included [mean age 43.8 (SD 15.9) years, 58% female, 48% with children, 68% with a partner, 25% with neither children nor partner]. Having children and/or a partner was associated with lowered willingness to trade life years translating to higher elicited health state utilities (*p* < 0.01).

**Conclusion:**

Having significant others, or the lack of having significant others, was associated with respondents’ valuation of hypothetical health states using TTO, more so than traditional sampling variables such as age and sex. Inadequate representativeness in terms of having significant others could bias health state preference values in valuation studies.

**Supplementary Information:**

The online version contains supplementary material available at 10.1007/s11136-021-03026-6.

## Plain English summary

We find that time trade-off (TTO) valuations for hypothetical health states depend notably on whether the respondent has children and/or a partner. Preferences to health are being included in health care decision-making as a way to measure outcomes in both length and quality of life. How, and from whom, these preferences are collected can impact the value of different health states. TTO is a commonly used method for valuing health, and the standard method for valuing the EQ-5D. The task involves respondents stating their preference between a shorter life in full health and a longer life in poor health. The findings suggest that valuation studies using TTO should aim to ensure representativeness in terms of having significant others, in order to avoid potential bias.

## Introduction

Health state valuation and the use of quality-adjusted life years (QALYs) have become integral elements in health economic evaluation and are increasingly in demand from policy makers in need of transparent and justifiable foundations for decisions in health care. Preference-based health state values reflecting the general population are a key component of QALYs, although preferences for particular age- or patient groups are also often collected.

Health state preferences are typically elicited using standard methods such as the time trade-off (TTO), standard gamble, discrete choice experiments, or visual analogue scales (VAS). Preferences for health depend on the respondents’ characteristics [1, 2], such as age, socio-economic status and educational level [3, 4]. Other factors also influence how people value health, particularly in TTO valuation, including perspectives on euthanasia [5] and religious views [6].

Standard protocols for eliciting population-values for the most commonly used instrument in health state valuation, the EQ-5D [7], recommend the use of the TTO in population-representative samples, though there is no specification of which characteristics the sample should reflect [8, 9]. Studies tend to focus on age, sex, and education, with representation of geographic regions [10–12], or ethnic subgroups [13] sometimes coming into play. Given that other individual characteristics may influence health preferences, taking these into account could arguably increase validity of values, and improve comparability between value sets and over time.

TTO values are based on a sequential process where the respondent is asked to state their preference for two alternative lives; in the simplest form, a shorter life in full health, and a longer life in a poorer health state (the state to be valued). The length of life in full health is modified based on respondent choices, until preferential indifference is reached, though respondents’ goals and priorities due to life circumstances could be influencing health preferences. A wish to live longer to see children grow up is an example of this.

Parenthood is a potentially life-changing transition, typically altering daily life and priorities. Studies have shown that parenthood and the transition into parenthood affects quality of life, with parents’ generally reporting lower quality of life, and risk aversion, with increased risk aversion present up to two years prior to parenthood [14, 15]. Though risk aversion may not play into the TTO task, other forms of bias may influence values differently for different groups, for example an overemphasis on time over health status. Parents value life years and health states differently using the TTO than the rest of the general population [1, 16, 17]. Studies assessing the effect of having a partner have been less conclusive, with some showing those with partners being more willing to trade life years, [1, 17], as well as a difference between being married and simply living together. Family related goals are important [18], goals for which respondents claim to be both willing to live a shorter life and in poorer health to attain. Inconclusive results in some studies are suggested to be a result of competing effects of having significant others, introducing the concept of "quality-of-life altruists" seeking to reduce the burden of one’s own poor health on loved ones [19], potentially cancelling out the effect of those with lowered willingness to trade.

Previous studies assessing the effect of significant others have generally focused on the valuation of mild health states, based on smaller data sets (n < 150) or with data collected online. Online data collection has been shown to give higher rates of clustered/extreme values than data collected in face-to-face interviews [20]. Health state valuation with TTO is demanding, with misunderstandings common without the guidance of an interviewer [21]. Latest protocols clearly favour data collections by face-to-face interview [8].

This study aimed to explore the effect of having significant others, hereunder having children and/or having a partner, on TTO valuations of hypothetical health states in a valuation study complying with the EuroQol valuation technology (EQ-VT) protocol [8]. We hypothesized that, using TTO, (1) individuals with children (< 18 years), (2) individuals with a live-in partner/spouse, or (3) any significant other (children or partner) would value health states differently than individuals without children and partner.

## Methods

### Study design and sample

The study used data from the Norwegian EQ-5D-5L valuation study. Data collection started in November 2019, but stopped in March 2020 due to the COVID-19 pandemic, at which point 542 interviews were completed. The study intentionally oversampled selected groups typically hard to reach, including ethnic minorities, those with lower socio-economic status and parents of young children [22].

Respondents were invited to the study via randomly sampled locations within different geographic areas in Norway and location type strata aimed at reaching different respondent groups. Contact persons at each location assisted, where feasible, to meet quotas according to gender and age. Child day-care facilities and primary schools were sampled to increase the number of respondents with young children.

### Interviews and questionnaires

Data were collected by one-to-one pc-assisted interviews, following standard EQ-VT protocol version 2.1 [9], and guided by a trained interviewer. See the original study protocol for more details on training and use of valuation technology [22]. Interviews were completed at sampled locations, for example libraries, schools, workplaces or recreational centres. Where possible, interviews were completed in separate rooms. Standard EuroQol quality controls (QC) were assessed throughout data collection [23], with flags related to time spent on the task, the introduction to lead-time TTO, and inconsistent valuations of the worst possible health state. Protocol compliance was found to be excellent, with few interviews flagged for poor data quality.

Interviews were conducted using the EQ-PVT, a portable version of the EQ-VT software developed by EuroQol. The EQ-PVT provides a similar visual presentation of the TTO tasks as the EQ-VT software, presented as two horizontal scales indicating number of years in Life A (a life in full health) and Life B (a life in the health state to be valued). The respondent values each health state by choosing between Life A and Life B in an iterative process until the respondent perceives the two lives to be of about the same value.

Following EQ-VT protocol, composite time trade-off (cTTO) was administered [8, 24]. The cTTO is a modified version of the TTO, where lead-time TTO is used for the valuation of states identified as worse than being dead (WTD). When states are judged to be WTD, the respondent is offered an additional 10 years in full health lead-time in Life B, a total of 20 years (10 years in full health, followed by 10 years in the health state to be valued), as an alternative to 10 or fewer years in full health in Life A.

Interviewers followed a standardised interview guide with scripted introduction and recommended responses, introducing all parts of the cTTO task, including the concept of WTD, and how to give such values using the cTTO. Respondents practiced by valuing three practice states before completing 10 cTTO valuations.

The interviewer guided the respondent through the entire interview and answered questions respondents had throughout. The interviewer was instructed to not comment on seemingly illogical responses, but to encourage respondents to think aloud and carefully consider each health state presented. After completing all TTO tasks, respondents were asked to review responses and flag any they deemed inconsistent in a feedback module, without comment from the interviewer.

In addition to the valuation tasks, each respondent defined their own health with the EQ-5D-5L and VAS, and completed a paper questionnaire with items describing their background. The standard EuroQol visual analogue scale (EQ VAS) from 0 to 100 was used, with 100 representing best imaginable health and 0 worst imaginable health. Respondents defined their own health state prior to completing valuation tasks, and to conclude completed the rest of the questionnaire, including questions about significant others.

Information on significant others was collected from questionnaire items where respondents indicated how many children under 18 years of age they had responsibility for, as well as their marital/partner status. The items were formulated as “Do you have responsibility for children under the age of 18?”, where respondents indicated the number of children for whom they were responsible, and “What is your marital status?”, with the response categories “Single”, “Married”, “Cohabiting”, “Divorced/separated”, “Widowed”. Responses for these items were recoded to “with children under 18 years of age” if they stated that they had responsibility for at least one child under 18 years of age, and with a partner if they indicated that they were either married or co-habiting.

### Statistical analysis

Descriptive statistics summarized respondent characteristics. Linear regression models assessed the association of the main analysis variables with use of the feedback module and QC flags.

Each respondent provided ten individual TTO valuations, all of which were included in the primary analyses, irrespective of flagging in the feedback module. To account for the nested nature of the data, a mixed model with a random intercept at the respondent level was used to estimate the effects of having significant others on willingness to trade. We used disutility (= 1-utility) in the analyses. Values elicited using the cTTO procedure are left-censored at − 1. Correspondingly, disutility values were handled as being right-censored at 2, i.e. a Tobit model. The effect of age on elicited values was explored prior to final modelling using descriptive methods and loess regression (Supplementary Fig. 1).

We tested five different models. Model 1 included only having children as a significant other, as well as age, sex, and higher education. Model 2 was similar to Model 1, but with a dummy variable for having a partner instead of children as significant other. Model 3 included both variables for having children and having a partner, and Model 4 included an interaction between the two. Model 5 included only a dummy variable for any significant other, indicating either children or a partner, in addition to age, sex and higher education, as in previous models. We defined dummy variables coded 1 for: individuals with children < 18 years (*CHILD*); individuals living with a partner or married (*PART)*; individuals with either children or a partner (*SIGNIF)*; female respondents (*FEM)*; individuals with higher education (*EDU).* Age in years was included as a continuous variable (AGE). For more flexible modelling and to account for the non-linear relationship of age and disutility, we made use of natural splines; a form of flexible interpolation by use of a pre-defined set of polynomials. In the equation, *ns* represents a function for cubic (3-knot) natural splines. Knots were placed at the quartiles of age in the data, giving four estimates in total. Final number of knots was determined by the Akaike information criterion. The five models were defined as following:

$${\text{Model 1: }}\,disutility \sim \alpha + \beta_{{ns\left( {AGE} \right)}} + \beta_{EDU} + \beta_{FEM} + \beta_{CHILD } + b_{0id}$$.

$${\text{Model 2: }}\,disutility \sim \alpha + \beta_{{ns\left( {AGE} \right)}} + \beta_{EDU} + \beta_{FEM} + \beta_{PART } + b_{0id}$$.

$${\text{Model 3: }}\,disutility \sim \alpha + \beta_{{ns\left( {AGE} \right)}} + \beta_{EDU} + \beta_{FEM} + \beta_{CHILD} + \beta_{PART } + b_{0id}$$.

$${\text{Model 4: }}\,disutility \sim \alpha + \beta_{{ns\left( {AGE} \right)}} + \beta_{EDU} + \beta_{FEM} + \beta_{CHILD} + \beta_{PART } + \beta_{CHILD:PART} + b_{0id}$$.

$${\text{Model 5: }}\,disutility \sim \alpha + \beta_{{ns\left( {AGE} \right)}} + \beta_{EDU} + \beta_{FEM} + \beta_{SIGNIF } + b_{0id}$$.

Sensitivity analyses controlled for interviewer effects, being married versus co-habiting, the health state valued, respondent’s self-reported health (EQ VAS), and included respondents with missing values for number of children, and excluded responses flagged in the feedback module. We coded missing values for children as not having indicated any children under the age of 18. To control for the health state we performed two analyses, including dummy variables per level per dimension of the health state, as well the level sum score (representing the deviation from full health) as a measure of the health state’s general severity.

R version 3.6.2 was used for the statistical analyses [25]. We chose a 5% significance level, using two-sided tests.

The Regional Committee for Medical and Research Ethics reviewed the study and stated that their approval was not required. The Norwegian Institute of Public Health approved the Data Protection Impact Assessment for the study 30^th^ September 2019.

## Results

### Sample characteristics

Of the 542 interviews completed, responses from 430 respondents were included, with 10 responses per respondent. Respondents with missing responses, either missing TTO responses (*n* = 31), completely missing paper questionnaire responses (*n* = 5), or missing response for item on number of children (*n* = 76), were excluded from the analyses. The mean age of the sample was 44 years; 58% of respondents were female, and 61% had completed higher education (Table 1). Almost half (48%) indicated having responsibility for at least one child under the age of 18 years, and 68% had a partner. In total, 25% of respondents indicated that they had neither children under the age of 18 nor a partner, whilst 35% indicated that they had both. Respondents had a mean VAS score of 78.8, where those with a partner, either with children (79.8) or without (80.7), scored their own health today as slightly higher than those without a partner (with children = 72.6, without children = 76.9).

**Table 1:** Sample demographics and EQ VAS score for total sample and those with significant others (with/without children and/or partner)

	Total	With children, no partner	With children, with partner	No children, no partner	No children, with partner
N	430	30	175	106	119
Age, mean (SD)	43.8 (15.9)	43.4 (9.5)	43.1 (9.2)	37.2 (20.7)	50.9 (17.1)
No. of women (%)	250 (58.1)	23 (76.7)	110 (62.6)	63 (59.4)	54 (45.4)
No. with higher education (%)	261 (60.7)	13 (43.3)	124 (70.8)	43 (40.6)	81 (68.1)
No. with children under 18 years (%)	205 (47.7)				
No. with partner (%)	294 (68.4)				
No. without children and partner (%)	106 (24.7)				
EQ VAS score, mean (SD)	78.8 (16.5)	72.6 (20.6)	79.8 (15.5)	76.9 (17.5)	80.7 (15.7)

### Data quality

Of included responses, 445 had been flagged by respondents in the feedback module as inconsistent [median 1 flagged per respondent, min 0 (*n* = 179), max 5 (*n* = 4)]. Fifteen interviews were flagged for data quality concerns; most of these (*n* = 9) were inconsistent valuations of the worst possible health state. Regression models showed no significant association between having significant others and use of the feedback module or QC flags (Supplementary Table 6).

### Time trade-off valuations

**Fig. 1:**
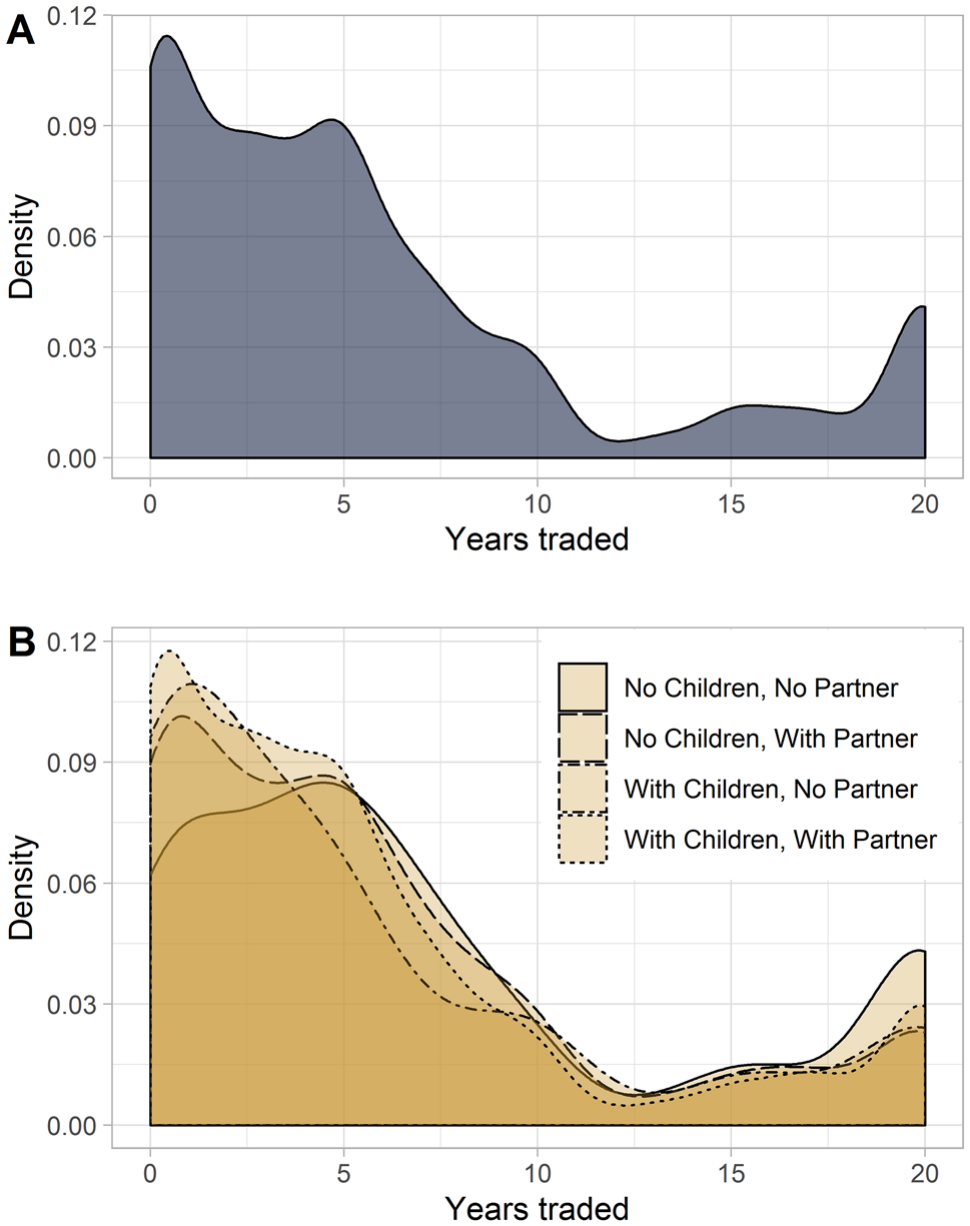
Number of years traded per TTO task for (**A**) the total sample (**B**) subgroups with/without children and/or partner

On average, respondents traded 5.9 years per health state. Those with neither children nor partner traded mean 7.2 years and those with both 5.4 years (Fig. 1). 335 observations were right-censored with a maximum disutility of 2. Respondents without significant others defined 22% of valuations as WTD (respondents with significant others: 15%). Logistic regression showed that those with a partner were less likely to value health states as WTD (*p* < 0.05) (Supplementary Table 7).

Both having children and having a partner were found to have a significant effect on willingness to trade (*p* < 0.05), with respondents with either a partner or at least one child under the age of 18 assigning lower disutility to presented health states (Table 2). All models included age, sex, and higher education as independent variables, and having significant others had a larger effect than of both sex and higher education.

**Table 2:** Estimated coefficients (standard error) from linear mixed models estimating disutility by respondents characteristics (having children under the age of 18, having a partner, age, sex and higher education)

	Model 1	Model 2	Model 3	Model 4	Model 5
Children	− 0.120**		− 0.086	− 0.249***	
	(0.049)		(0.050)	(0.088)	
Partner		− 0.173***	− 0.156***	− 0.234***	
		(0.046)	(0.047)	(0.058)	
Children × partner				0.216**	
				(0.097)	
Significant other					− 0.248***
					(0.053)
ns(Age)1	0.085	0.057	0.152	0.176*	0.150
	(0.104)	(0.090)	(0.104)	(0.104)	(0.094)
ns(Age)2	− 0.087	0.019	− 0.012	0.056	0.074
	(0.099)	(0.099)	(0.100)	(0.104)	(0.100)
ns(Age)3	0.369*	0.477**	0.525***	0.635***	0.634***
	(0.192)	(0.193)	(0.195)	(0.200)	(0.200)
ns(Age)4	0.427***	0.416***	0.415***	0.386***	0.385***
	(0.152)	(0.149)	(0.149)	(0.148)	(0.148)
Female	0.067*	0.048	0.051	0.055	0.055
	(0.040)	(0.040)	(0.040)	(0.039)	(0.039)
Higher education	0.016	0.046	0.042	0.037	0.037
	(0.041)	(0.041)	(0.041)	(0.041)	(0.040)
Constant	0.476***	0.520***	0.504***	0.499***	0.502***
	(0.094)	(0.093)	(0.093)	(0.092)	(0.092)
Observations	4300	4300	4300	4300	4300
Right-censored	335	335	335	335	335
Log likelihood	− 3924.449	− 3920.485	− 3918.987	− 3916.533	− 3916.729
Akaike information criterion	7864.899	7856.971	7855.973	7853.065	7849.459

Random intercept included at respondent level, values right-censored at disutility = 2. Interaction between having children and having a partner included in Model 4. Significant other in Model 5 representing having either children or a partner. Age modelled using natural splines (ns) with knots at the quartiles of age giving estimates for ns(Age)1–4

*p < 0.1; **p < 0.05; ***p < 0.01

**Fig. 2:**
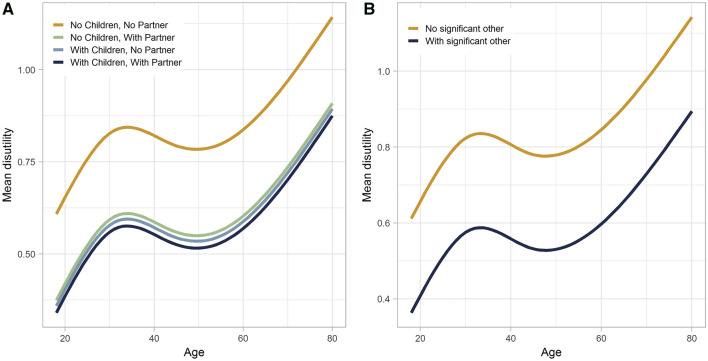
Mean disutility per TTO task by age for (**A**) populations with/without children under the age of 18 and/or partner and (**B**) population with/without significant other (children or partner), predictions based on final models (model 4 and 5)

Predicted values based on the final models indicated that those without any significant other were on average willing to trade at least two more life years than those with significant others (Fig. 2).

### Sensitivity analyses

Sensitivity analyses showed that both being married and co-habiting with a partner reduced willingness to trade, though more so for respondents who were married (*p* < 0.01) (Supplementary Table 1). Controlling for the interviewer did not change the effect of significant others (Supplementary Table 3). Further, analyses were repeated controlling for the health state valued, and the health state of the respondent (Supplementary Table 2, 8). Poorer respondent health did not have a significant effect on willingness to trade, in a univariate regression analysis or adjusted for the other covariates.

The effect of having children and/or a partner remained statistically significant in all sensitivity analyses and with comparable effect size as found in the main analyses. Findings were robust after excluding responses flagged in the feedback module (Supplementary Table 5). Including those with missing values for number of children, resulted in a significant though slightly reduced effect of having significant others (Supplementary Table 4). Analyses exploring how best to model age, showed that the effect of age seemed to depend on whether the respondent had significant others (Supplementary Fig. 1). Analyses without a random intercept at the respondent level resulted in a significant interaction between age and partner, where those with a partner valued health states with lower disutility even in older age. This interaction was not statistically significant when the random intercept was included and was not included in the final models.

## Discussion

This study has shown that having significant others, here defined as children under the age of 18 and/or a partner, was associated with the disutility assigned to health states, i.e. the number of years respondents were willing to trade in a TTO. Having children, a partner, or both, all showed a similar association. Models including both having children and having a partner (models 3 and 4) suggest that having a partner, more so than children, was driving the effect of significant others. Respondents with a partner were also less likely to value health states as WTD. Not having significant others increased the number of years the respondent was willing to trade by approximately 2 years on average, resulting in greater disutility scores.

Previous studies have found similar associations between having children [16, 26] or having a partner [1, 19] and willingness to trade. Qualitative interviews with mothers after completing TTO implied that willingness to trade may not be as easily explained as target life expectancy [26]. The similar effect of being a parent, having a partner, and the interaction between the two, may be an expression of this. One previous study found conflicting effects for those married and those co-habiting with a partner [17]. Sensitivity analyses in the present study distinguishing between being married and co-habiting did not support this, though it should be noted that this may be culture-specific, as the difference between co-habitation and being married is not strongly emphasized in Norway. We did not attempt to assess the direction of causality between having significant others and willingness to trade and cannot rule out that individuals more willing to trade may be less likely to have significant others.

Previous studies assessing the effect of having significant others have largely focused on the valuation of milder health states. Following EQ-VT protocol, we asked respondents to value health states ranging from the mildest to the most severe, with lead-time TTO for WTD valuation. The use of lead-time TTO potentially complicates the interpretation of reduced willingness to trade as a wish to maximise time with loved ones, given that lead-time offers extended life duration.

Our results could seem to indicate greater willingness to trade life years with increasing respondent age. However, early analyses, without random effects, suggested an interaction between age and that of having a partner on willingness to trade, which to our knowledge is unexplored in other studies. Respondents with a partner seemed to be less willing to trade life years than respondents without, even in older age (Supplementary Fig. 1). As respondents got older, an increasing proportion identified themselves as without a partner. Previously found increasing willingness to trade with age, for example [27], could thus in part be attributable to respondents increasingly living without partners as they get older. With the inclusion of the random intercept at the respondent level, the interaction between age and having a partner did not reach statistical significance.

We have chosen to focus only on TTO valuations, and, as has also been suggested to be the case for religious respondents [28], difference in values can be interpreted as an artefact of the TTO itself. Matza et. al. found that caregiver status seemed to have less impact on willingness to gamble in standard gamble than willingness to trade in TTO [16]. Differences between TTO and standard gamble values can be attributed to differences in bias, for example with TTO more prone to bias from scale compatibility [29]. Subjective expectations to length and quality of life have been shown to influence TTO valuations and willingness to trade life years [30], and though having significant others may not lead to differing expectations for length of life, it could lead to differing expectations to future quality of life.

Observed differences in willingness to trade life years indicate that TTO is sensitive to factors beyond the severity of the health states intended for valuation. This can be interpreted in at least two different ways. On the one hand, the aim of health state valuation is to gain a measure of the how (dis)preferable the health states to be valued are, with emphasis on the preference for health states in isolation. Arguably, sensitivity to factors beyond the qualities of the particular health state in question should be out of scope. Alternatively, one could argue that the presence of significant others could have a real and valid impact on preferences for time alive, the denominator in TTO. From this perspective, the observed sensitivity to life situation could be an indication of validity. Regardless of perspective, the findings suggest that studies aiming to produce population-representative preference values should take measures to ensure representative sample in terms of the proportion of respondents with significant others.

### Strengths and limitations

A strength of this study was the use of trained interviewers and face-to-face personal interviews. Interviewer training is important because the TTO can be difficult to comprehend and complete without interviewer guidance [20, 21]. Respondents included in this study completed TTO tasks before indicating their marital status and number of children under the age of 18. Therefore, interviewers were blinded to this information during the TTO exercises.

Some limitations should be noted. Respondents indicated only number of children under the age of 18 years for whom they had responsibility, and we had no information about older children, grandchildren, or other children in their family. In 2020, average age for first-time parenthood in Norway was approximately 30 years (mean age of new mothers 29.9 years; new fathers 32.1) [31]. Not surprisingly, only one respondent under the age of 25 years, and few respondents over the age of 65 (*n* = 4), indicated having responsibility for a child under the age of 18. Older respondents may however have grandchildren or older children, which could be influencing their responses. A considerable number of respondents (*n* = 76) chose not to answer the survey item for children, and one could speculate as to the reason for this. Respondents with children over the age of 18 years, or those without children may have deemed the item irrelevant, or they may have had children but not wished to answer, thus muddying the results when this group was included. Mean disutility per health state was lower for respondents in middle age (e.g. 40–60 years of age), as shown by a decrease in years traded for all groups including those without significant others. Given the formulation of the questionnaire item for having children and the restriction to children under the age of 18, lower willingness to trade in this age group may be an expression of respondents typically having older children at this age, which would be not captured being by the survey item.

Respondents indicated their marital status, with single, married, divorced, co-habiting, and widow as response options, yet significant others may fall outside the categories provided, for example those living with other family members. The study included no information on the age of the children in question. Without the age, it is not possible to explore whether the effect of being a parent on willingness to trade changes over time, or is a passing effect, as has been suggested by some studies arguing that the effect of children may be an intrinsic effect, or, as with risk aversion, present prior to parenthood then decreasing over time [15].

## Implications of results

Representative samples are imperative in valuation studies, and studies attempt to ensure samples mirroring the population from which they come. The focus of sampling strategies have traditionally been age, sex, level of education, and in some studies, geography, ethnicity and religion. The results from this study suggest that having significant others, or the lack thereof, potentially has a greater effect on respondents’ valuation of health states using TTO than traditional sampling variables, such as age and sex. Though this study cannot determine the direction of causality between the two, the implications of a clear association between having significant others and willingness to trade do not change.

Establishing a representative sample is contingent on available information describing the population according the variables in question. In Norway, individual level family and household type are amongst variables readily available to researchers [32]. Similar statistics are also available in other countries, for example in the UK and USA [33, 34]. Including potential respondents by family status specifically may not be as straightforward, but can be addressed by choice of sampling strategy, for example stratified or quota sampling, or by taking family into account in the statistical analyses by weighting responses to better reflect the population. Based on the magnitude of impact of having children or a partner, future valuation studies should consider including such characteristics to ensure representativeness in terms of variables of true importance for health preferences.

## Supplementary Information

Below is the link to the electronic supplementary material.

Supplementary file1 (DOCX 502 kb)

## Data Availability

Raw data cannot be shared due to privacy laws in Norway.

## Abbreviations

EQ-VT: EuroQol valuation technology
EQ-PVT: EuroQol portable valuation technology
(EQ) VAS: (EuroQol) visual analogue scale
QALY: Quality adjusted life year
QC: Quality control
(c)TTO: (Composite) time trade-off
WTD: Worse than being dead

## Code availability

Code can be shared upon reasonable request to the corresponding author.
