# Correction to: Time trade‑off with someone to live for: impact of having significant others on time trade‑off valuations of hypothetical health states

**DOI:** 10.1007/s11136-022-03160-9

**Published:** 2022-05-18

**Authors:** Tonya Moen Hansen, Knut Stavem, Kim Rand

**Affiliations:** 1 Division for Health Services, Norwegian Institute of Public Health, Oslo, Norway; 2 Health Services Research Unit, Akershus University Hospital, Nordbyhagen, Norway; 3 Institute of Clinical Medicine, University of Oslo, Oslo, Norway; 4 Medical Division, Department of Pulmonary Medicine, Akershus University Hospital, Lørenskog, Norway; 5 Maths in Health B.V, Rotterdam, The Netherlands

## Correction to: Quality of Life Research (2022) 31:1199–1207 10.1007/s11136-021-03026-6

In the original publication, one of the affiliation information was inadvertently missed for the corresponding author Tonya Moen Hansen. It is updated in this correction.

The original article has been corrected.

