# Supplementary material for: Time trade-off with someone to live for: impact of having significant others on time trade-off valuations of hypothetical health states
Source: Qual Life Res. 2021 Oct 30;31(4):1199–207. doi: 10.1007/s11136-021-03026-6 (PMC8556854; doi:10.1007/s11136-021-03026-6)
Supplement: Supplementary file 1 — Supplementary file1 (DOCX 502 kb) [file 11136_2021_3026_MOESM1_ESM.docx]

## Supplementary Material

**Supplementary Figure 1:** Mean number of years traded by age for (A) all respondents, respondents (B) with/without a partner, (C) with/without children under the age of 18 and (D) with/no significant other (either children or partner), smooth curve fitted using loess regression (years traded ~ age) with 95% CI

### Results from sensitivity analyses

**Supplementary Table 1:** Estimated coefficients (standard error) from linear mixed models exploring the association between disutility and *having a partner (Model 1 & 3), being married and cohabiting (Model 2 & 4), and including interactions with having children (Model 3, 5-6)*. Dependent variable: disutility. Random intercept included at the respondent level. Age modelled using natural splines (ns) with knots at the quartiles of age giving estimates for ns(Age)1-4.

**Supplementary Table 2:** Estimated coefficients (standard error) from linear mixed models exploring the association between disutility and respondent health status, both as univariate association between disutility and health status (Model 1 numerical EQ VAS score, Model 2 EQ-VAS score group), and Models 3-5 adjusted for other variables (significant others, age, sex and higher education) . Dependent variable: disutility. Random intercept included at the respondent level. Age modelled using natural splines (ns) with knots at the quartiles of age giving estimates for ns(Age)1-4.

**Supplementary Table 3:** Estimated coefficients (standard error) from linear mixed models. Dependent variable: disutility. Nested random intercepts at the level of respondents within interviewers. Age modelled using natural splines (ns) with knots at the quartiles of age giving estimates for ns(Age)1-4.

**Supplementary Table 4:** Estimated coefficients (standard error) from linear mixed models, for *final sample and sample including respondents with missing item response for “responsibility for children under 18 years”.* Dependent variable: disutility. Random intercept included at the respondent level. Age modelled using natural splines (ns) with knots at the quartiles of age giving estimates for ns(Age)1-4.

**Supplementary Table 5:** Estimated coefficients (standard error) from linear mixed models. *Health states flagged in the feedback module excluded*. Dependent variable: disutility. Random intercept included at the respondent level. Age modelled using natural splines (ns) with knots at the quartiles of age giving estimates for ns(Age)1-4.

**Supplementary Table 6:** Estimated coefficients (standard error) from linear regression models estimating *number of states flagged in the feedback module per respondent (Model 1) and number of quality control flags per respondent (Model 2) by respondent characteristics*.

**Supplementary Table 7:** Estimated coefficients (standard error) from generalized linear mixed model estimating association between *valuing a health state as “Worse than being dead”* and respondent characteristics (having children/partner/both, age, sex, higher education). Random intercept included at both the interviewer and respondent level. Age modelled using natural splines (ns) with knots at the quartiles of age giving estimates for ns(Age)1-4.

**Supplementary Table 8:** Estimated coefficients (standard error) from linear mixed model estimating disutility adjusted for the severity of the health state (here defined by the health states Level Sum Score (LSS)/deviation from full health) and respondent characteristics (having children/partner/both, age, sex, higher education). Random intercept included at both the interviewer and respondent level. Age modelled using natural splines (ns) with knots at the quartiles of age giving estimates for ns(Age)1-4.

**Supplementary Figure 1:** Mean number of years traded by age for (A) all respondents, respondents (B) with/without a partner, (C) with/without children under the age of 18 and (D) with/no significant other (either children or partner), smooth curve fitted using loess regression (years traded ~ age) with 95% CI


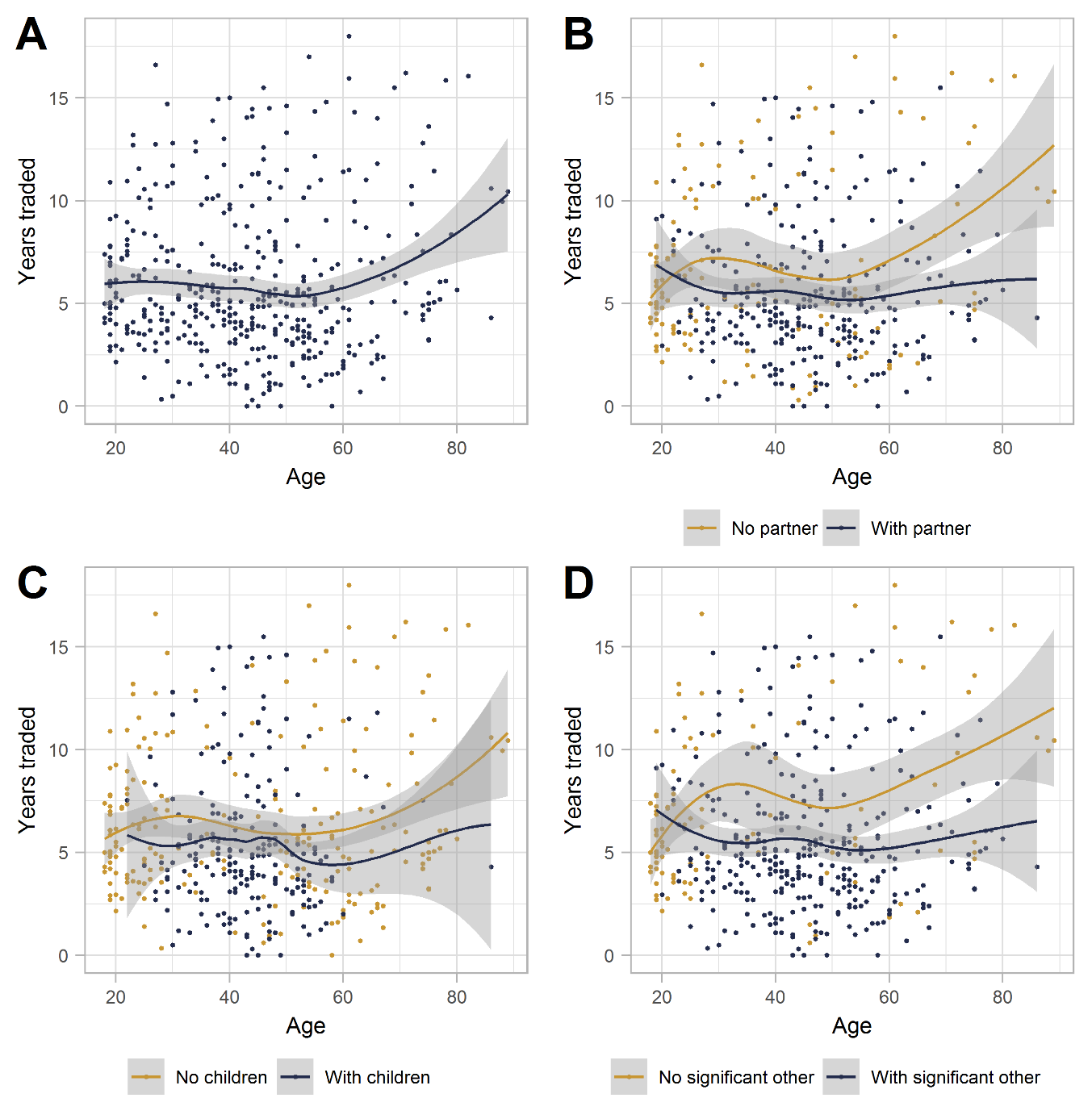


**Supplementary Table 1:** Estimated coefficients (standard error) from linear mixed models exploring the association between disutility and *having a partner (Model 1 & 3), being married and cohabiting (Model 2 & 4), and including interactions with having children (Model 3, 5-6)*. Dependent variable: disutility. Random intercept included at the respondent level. Age modelled using natural splines (ns) with knots at the quartiles of age giving estimates for ns(Age)1-4.

|  | Model 1 | Model 2 | Model 3 | Model 4 | Model 5 | Model 6 |
| --- | --- | --- | --- | --- | --- | --- |
|  |  |  |  |  |  |  |
| Children |  |  | -0.216*** | -0.075 | -0.120** | -0.092* |
|  |  |  | (0.081) | (0.046) | (0.060) | (0.050) |
|  |  |  |  |  |  |  |
| Partner | -0.145*** |  | -0.196*** |  |  |  |
|  | (0.042) |  | (0.053) |  |  |  |
|  |  |  |  |  |  |  |
| Married |  | -0.161*** |  | -0.143*** | -0.188*** | -0.136*** |
|  |  | (0.046) |  | (0.047) | (0.061) | (0.048) |
|  |  |  |  |  |  |  |
| Cohabiting |  | -0.118** |  | -0.109** | -0.095* | -0.142** |
|  |  | (0.051) |  | (0.052) | (0.053) | (0.066) |
|  |  |  |  |  |  |  |
| Children *×* Partner |  |  | 0.183** |  |  |  |
|  |  |  | (0.088) |  |  |  |
|  |  |  |  |  |  |  |
| Children *×* Married |  |  |  |  | 0.091 |  |
|  |  |  |  |  | (0.078) |  |
|  |  |  |  |  |  |  |
| Children *×* Cohabiting |  |  |  |  |  | 0.069 |
|  |  |  |  |  |  | (0.087) |
|  |  |  |  |  |  |  |
| ns(Age)1 | 0.032 | 0.043 | 0.138 | 0.123 | 0.140 | 0.119 |
|  | (0.082) | (0.083) | (0.096) | (0.096) | (0.097) | (0.096) |
|  |  |  |  |  |  |  |
| ns(Age)2 | -0.014 | 0.001 | 0.015 | -0.028 | 0.005 | -0.030 |
|  | (0.090) | (0.092) | (0.095) | (0.094) | (0.098) | (0.094) |
|  |  |  |  |  |  |  |
| ns(Age)3 | 0.395** | 0.406** | 0.531*** | 0.447** | 0.498*** | 0.443** |
|  | (0.177) | (0.177) | (0.183) | (0.178) | (0.184) | (0.179) |
|  |  |  |  |  |  |  |
| ns(Age)4 | 0.391*** | 0.405*** | 0.365*** | 0.401*** | 0.410*** | 0.386*** |
|  | (0.136) | (0.137) | (0.136) | (0.137) | (0.137) | (0.138) |
|  |  |  |  |  |  |  |
| Female | 0.037 | 0.036 | 0.044 | 0.039 | 0.042 | 0.038 |
|  | (0.036) | (0.036) | (0.036) | (0.036) | (0.036) | (0.036) |
|  |  |  |  |  |  |  |
| Higher education | 0.042 | 0.041 | 0.035 | 0.037 | 0.031 | 0.040 |
|  | (0.037) | (0.037) | (0.037) | (0.037) | (0.038) | (0.038) |
|  |  |  |  |  |  |  |
| Constant | 0.531*** | 0.529*** | 0.512*** | 0.515*** | 0.507*** | 0.520*** |
|  | (0.085) | (0.085) | (0.085) | (0.085) | (0.085) | (0.085) |
|  |  |  |  |  |  |  |
| Observations | 4,300 | 4,300 | 4,300 | 4,300 | 4,300 | 4,300 |
| Log Likelihood | -3,483.229 | -3,484.953 | -3,483.300 | -3,485.778 | -3,486.737 | -3,486.989 |
| Akaike information criterion | 6,986.459 | 6,991.905 | 6,990.600 | 6,995.557 | 6,999.475 | 6,999.978 |
| Note: *p<0.1; **p<0.05; ***p<0.01 |  |  |  |  |  |  |

**Supplementary Table 2:** Estimated coefficients (standard error) from linear mixed models exploring the association between disutility and respondent health status, both as univariate association between disutility and health status (Model 1 numerical EQ VAS score, Model 2 EQ-VAS score group), and Models 3-5 adjusted for other variables (significant others, age, sex and higher education) . Dependent variable: disutility. Random intercept included at the respondent level. Age modelled using natural splines (ns) with knots at the quartiles of age giving estimates for ns(Age)1-4.

|  | Model 1 | Model 2 | Model 3 | Model 4 | Model 5 |
| --- | --- | --- | --- | --- | --- |
| Respondents EQ VAS score | -0.001 |  | -0.0004 |  |  |
|  | (0.001) |  | (0.001) |  |  |
|  |  |  |  |  |  |
| EQ-VAS score = 25-49 |  | 0.179 |  | 0.172 |  |
|  |  | (0.199) |  | (0.196) |  |
|  |  |  |  |  |  |
| EQ-VAS score = 50-74 |  | 0.099 |  | 0.116 |  |
|  |  | (0.185) |  | (0.182) |  |
|  |  |  |  |  |  |
| EQ-VAS score = 75-100 |  | 0.049 |  | 0.075 |  |
|  |  | (0.182) |  | (0.179) |  |
|  |  |  |  |  |  |
| Respondent EQ-VS score below sample average EQ-VAS score (78.8) |  |  |  |  | -0.016 |
|  |  |  |  |  | (0.036) |
|  |  |  |  |  |  |
| Significant other (partner or children) |  |  | -0.208*** | -0.188*** | -0.213*** |
|  |  |  | (0.049) | (0.051) | (0.049) |
|  |  |  |  |  |  |
| ns(Age)1 |  |  | 0.108 | 0.068 | 0.115 |
|  |  |  | (0.087) | (0.090) | (0.087) |
|  |  |  |  |  |  |
| ns(Age)2 |  |  | 0.027 | 0.003 | 0.037 |
|  |  |  | (0.093) | (0.098) | (0.092) |
|  |  |  |  |  |  |
| ns(Age)3 |  |  | 0.520*** | 0.536*** | 0.531*** |
|  |  |  | (0.183) | (0.189) | (0.182) |
|  |  |  |  |  |  |
| ns(Age)4 |  |  | 0.366*** | 0.413*** | 0.359*** |
|  |  |  | (0.136) | (0.150) | (0.136) |
|  |  |  |  |  |  |
| Female |  |  | 0.042 | 0.033 | 0.044 |
|  |  |  | (0.036) | (0.037) | (0.036) |
|  |  |  |  |  |  |
| Higher education |  |  | 0.038 | 0.043 | 0.033 |
|  |  |  | (0.037) | (0.038) | (0.037) |
|  |  |  |  |  |  |
| Constant | 0.645*** | 0.524*** | 0.554*** | 0.441** | 0.521*** |
|  | (0.085) | (0.181) | (0.123) | (0.202) | (0.084) |
|  |  |  |  |  |  |
| Observations | 4,300 | 4,300 | 4,300 | 4,300 | 4,300 |
| Log Likelihood | -3,490.867 | -3,275.123 | -3,485.540 | -3,270.336 | -3,481.991 |
| Akaike information criterion | 6,989.734 | 6,562.247 | 6,993.080 | 6,566.672 | 6,985.981 |
| Note: *p<0.1; **p<0.05; ***p<0.01 | |  |  |  |  |

**Supplementary Table 3:** Estimated coefficients (standard error) from linear mixed models. Dependent variable: disutility. Nested random intercepts at the level of respondents within interviewers. Age modelled using natural splines (ns) with knots at the quartiles of age giving estimates for ns(Age)1-4.

|  | Model 1 | Model 2 | Model 3 | Model 4 | Model 5 |
| --- | --- | --- | --- | --- | --- |
|  |  |  |  |  |  |
| Children | -0.091* |  | -0.067 | -0.219*** |  |
|  | (0.048) |  | (0.049) | (0.083) |  |
|  |  |  |  |  |  |
| Partner |  | -0.105** | -0.090** | -0.154*** |  |
|  |  | (0.043) | (0.044) | (0.052) |  |
|  |  |  |  |  |  |
| Children *×* Partner |  |  |  | 0.204** |  |
|  |  |  |  | (0.090) |  |
|  |  |  |  |  |  |
| Significant other |  |  |  |  | -0.165*** |
|  |  |  |  |  | (0.046) |
|  |  |  |  |  |  |
| ns(Age)1 | 0.122 | 0.094 | 0.163* | 0.171* | 0.158* |
|  | (0.095) | (0.082) | (0.096) | (0.095) | (0.085) |
|  |  |  |  |  |  |
| ns(Age)2 | 0.212** | 0.258*** | 0.240*** | 0.291*** | 0.292*** |
|  | (0.090) | (0.089) | (0.090) | (0.092) | (0.089) |
|  |  |  |  |  |  |
| ns(Age)3 | 0.257 | 0.267 | 0.312* | 0.381** | 0.367** |
|  | (0.174) | (0.172) | (0.175) | (0.176) | (0.174) |
|  |  |  |  |  |  |
| ns(Age)4 | -0.127 | -0.142 | -0.143 | -0.176 | -0.173 |
|  | (0.120) | (0.119) | (0.119) | (0.119) | (0.118) |
|  |  |  |  |  |  |
| Female | 0.038 | 0.016 | 0.021 | 0.025 | 0.020 |
|  | (0.038) | (0.038) | (0.038) | (0.038) | (0.037) |
|  |  |  |  |  |  |
| Higher education | 0.003 | 0.029 | 0.023 | 0.018 | 0.025 |
|  | (0.037) | (0.038) | (0.038) | (0.038) | (0.037) |
|  |  |  |  |  |  |
| Constant | 1.541*** | 1.590*** | 1.573*** | 1.573*** | 1.585*** |
|  | (0.085) | (0.085) | (0.086) | (0.085) | (0.083) |
|  |  |  |  |  |  |
| Observations | 706 | 706 | 706 | 706 | 706 |
| Log Likelihood | 51.295 | 52.336 | 51.163 | 52.200 | 55.591 |
| Akaike information criterion | -80.590 | -82.672 | -78.325 | -78.401 | -89.181 |
| Note: *p<0.1; **p<0.05; ***p<0.01 | |  |  |  |  |

**Supplementary Table 4:** Estimated coefficients (standard error) from linear mixed models, for *final sample and sample including respondents with missing item response for “responsibility for children under 18 years”.* Dependent variable: disutility. Random intercept included at the respondent level. Age modelled using natural splines (ns) with knots at the quartiles of age giving estimates for ns(Age)1-4.

|  | Final sample | Sample incl. respondents w/missing child response |
| --- | --- | --- |
|  |  |  |
| Children | -0.216*** | -0.201*** |
|  | (0.081) | (0.077) |
|  |  |  |
| Children *×* Partner | 0.183** | 0.154* |
|  | (0.088) | (0.085) |
|  |  |  |
| Partner | -0.196*** | -0.167*** |
|  | (0.053) | (0.047) |
|  |  |  |
| ns(Age)1 | 0.138 | 0.165* |
|  | (0.096) | (0.085) |
|  |  |  |
| ns(Age)2 | 0.015 | -0.023 |
|  | (0.095) | (0.090) |
|  |  |  |
| ns(Age)3 | 0.531*** | 0.509*** |
|  | (0.183) | (0.164) |
|  |  |  |
| ns(Age)4 | 0.365*** | 0.344** |
|  | (0.136) | (0.141) |
|  |  |  |
| Female | 0.044 | 0.047 |
|  | (0.036) | (0.033) |
|  |  |  |
| Higher education | 0.035 | 0.029 |
|  | (0.037) | (0.034) |
|  |  |  |
| Constant | 0.512*** | 0.487*** |
|  | (0.085) | (0.076) |
|  |  |  |
| Observations | 4,300 | 5,060 |
| Log Likelihood | -3,483.300 | -4,110.030 |
| Akaike information criterion | 6,990.600 | 8,244.059 |
| Note: *p<0.1; **p<0.05; ***p<0.01 | |  |

**Supplementary Table 5:** Estimated coefficients (standard error) from linear mixed models. *Health states flagged in the feedback module excluded*. Dependent variable: disutility. Random intercept included at the respondent level. Age modelled using natural splines (ns) with knots at the quartiles of age giving estimates for ns(Age)1-4.

|  | Model 1 | Model 2 | Model 3 | Model 4 | Model 5 |
| --- | --- | --- | --- | --- | --- |
|  |  |  |  |  |  |
| Children | -0.096** |  | -0.067 | -0.216*** |  |
|  | (0.046) |  | (0.046) | (0.082) |  |
|  |  |  |  |  |  |
| Partner |  | -0.147*** | -0.134*** | -0.206*** |  |
|  |  | (0.043) | (0.043) | (0.054) |  |
|  |  |  |  |  |  |
| Children *×* Partner |  |  |  | 0.196** |  |
|  |  |  |  | (0.089) |  |
|  |  |  |  |  |  |
| Significant other |  |  |  |  | -0.214*** |
|  |  |  |  |  | (0.049) |
|  |  |  |  |  |  |
| ns(Age)1 | 0.049 | 0.031 | 0.106 | 0.129 | 0.113 |
|  | (0.096) | (0.083) | (0.097) | (0.097) | (0.088) |
|  |  |  |  |  |  |
| ns(Age)2 | -0.070 | 0.018 | -0.006 | 0.056 | 0.066 |
|  | (0.091) | (0.092) | (0.093) | (0.097) | (0.093) |
|  |  |  |  |  |  |
| ns(Age)3 | 0.335* | 0.427** | 0.466** | 0.564*** | 0.563*** |
|  | (0.178) | (0.180) | (0.181) | (0.186) | (0.185) |
|  |  |  |  |  |  |
| ns(Age)4 | 0.423*** | 0.413*** | 0.411*** | 0.385*** | 0.384*** |
|  | (0.138) | (0.137) | (0.137) | (0.137) | (0.136) |
|  |  |  |  |  |  |
| Female | 0.061* | 0.046 | 0.048 | 0.052 | 0.052 |
|  | (0.037) | (0.037) | (0.037) | (0.036) | (0.036) |
|  |  |  |  |  |  |
| Higher education | 0.024 | 0.050 | 0.046 | 0.042 | 0.042 |
|  | (0.038) | (0.038) | (0.038) | (0.038) | (0.037) |
|  |  |  |  |  |  |
| Constant | 0.468*** | 0.505*** | 0.493*** | 0.488*** | 0.490*** |
|  | (0.087) | (0.086) | (0.086) | (0.086) | (0.085) |
|  |  |  |  |  |  |
| Observations | 3,846 | 3,846 | 3,846 | 3,846 | 3,846 |
| Log Likelihood | -3,202.617 | -3,199.010 | -3,200.100 | -3,199.198 | -3,195.461 |
| Akaike information criterion | 6,425.234 | 6,418.021 | 6,422.200 | 6,422.395 | 6,410.922 |
| Note: *p<0.1; **p<0.05; ***p<0.01 | |  |  |  |  |

**Supplementary Table 6:** Estimated coefficients (standard error) from linear regression models estimating *number of states flagged in the feedback module per respondent (Model 1) and number of quality control flags per respondent (Model 2) by respondent characteristics*.

|  | Model 1 | Model 2 |
| --- | --- | --- |
| Children | -0.068 | -0.007 |
|  | (0.119) | (0.020) |
|  |  |  |
| Partner | -0.009 | -0.035 |
|  | (0.134) | (0.022) |
|  |  |  |
| Age | -0.009** | -0.0005 |
|  | (0.004) | (0.001) |
|  |  |  |
| Female | 0.048 | 0.022 |
|  | (0.115) | (0.019) |
|  |  |  |
| Higher Education | -0.070 | 0.003 |
|  | (0.119) | (0.020) |
|  |  |  |
| Constant | 1.440*** | 0.049 |
|  | (0.262) | (0.044) |
|  |  |  |
| Observations | 429 | 429 |
| R2 | 0.020 | 0.016 |
| Adjusted R2 | 0.009 | 0.004 |
| Residual Std. Error | 1.132 (df = 423) | 0.189 (df = 423) |
| F Statistic | 1.748 (df = 5; 423) | 1.383 (df = 5; 423) |

**Supplementary Table 7:** Estimated coefficients (standard error) from generalized linear mixed model estimating association between *valuing a health state as “Worse than being dead”* and respondent characteristics (having children/partner/both, age, sex, higher education). Random intercept included at both the interviewer and respondent level. Age modelled using natural splines (ns) with knots at the quartiles of age giving estimates for ns(Age)1-4.

|  | «Worse than being dead» value |
| --- | --- |
| Children | -0.936* |
|  | (0.492) |
|  |  |
| Partner | -0.713** |
|  | (0.320) |
|  |  |
| Children × Partner | 0.746 |
|  | (0.539) |
|  |  |
| ns(Age)1 | 1.011* |
|  | (0.583) |
|  |  |
| ns(Age)2 | 0.037 |
|  | (0.576) |
|  |  |
| ns(Age)3 | 2.716** |
|  | (1.099) |
|  |  |
| ns(Age)4 | 2.119*** |
|  | (0.784) |
|  |  |
| Female | 0.376* |
|  | (0.223) |
|  |  |
| Higher education | -0.201 |
|  | (0.227) |
|  |  |
| Constant | -3.250*** |
|  | (0.545) |
|  |  |
| Observations | 4,3 |
| Log Likelihood | -1,597.459 |
| Akaike information criterion | 3,218.918 |
| Note:*p<0.1; **p<0.05; ***p<0.01 | |

**Supplementary Table 8:** Estimated coefficients (standard error) from linear mixed model estimating disutility adjusted for the severity of the health state (here defined by the health states Level Sum Score (LSS)/deviation from full health) and respondent characteristics (having children/partner/both, age, sex, higher education). Random intercept included at both the interviewer and respondent level. Age modelled using natural splines (ns) with knots at the quartiles of age giving estimates for ns(Age)1-4.

|  | Disutility |
| --- | --- |
| Children | -0.226*** |
|  | (0.079) |
|  |  |
| Partner | -0.188*** |
|  | (0.052) |
|  |  |
| Children × Partner | 0.185** |
|  | (0.086) |
|  |  |
| LevelSumScore | 0.063*** |
|  | (0.001) |
|  |  |
| ns(Age)1 | 0.158* |
|  | (0.093) |
|  |  |
| ns(Age)2 | -0.013 |
|  | (0.093) |
|  |  |
| ns(Age)3 | 0.543*** |
|  | (0.179) |
|  |  |
| ns(Age)4 | 0.405*** |
|  | (0.133) |
|  |  |
| Female | 0.041 |
|  | (0.035) |
|  |  |
| Higher education | 0.035 |
|  | (0.036) |
|  |  |
| Constant | -0.431*** |
|  | (0.088) |
|  |  |
| Observations | 4,3 |
| Log Likelihood | -2,169.173 |
| Akaike information criterion | 4,366.345 |
| Note: *p<0.1; **p<0.05; ***p<0.01 | |
